# Cardiac MRI-guided therapeutics in ischemic cardiomyopathy: impact on heart-team consensus and outcomes

**DOI:** 10.3389/fcvm.2026.1783962

**Published:** 2026-09-04

**Authors:** Deepak Reddy Karumuri, Jebaraj Rathinasamy, Anant Akash Sakthivel, Harikaran Reddy Ragireddy, Rizwan Suliankatchi Abdulkader, Ashish H. Shah, Vadivelu Ramalingam, Ramprassath Muthampatti Siddhan, Salman Salahuddin, Bijoy Jacob, Sathish Kumar Parasuraman, Cha Rajakaruna, Ravindran Rajendran, Srikanth Bhumana, Antony Wilson, Hari Lalith Kovvuri, Pavitraa Saravana Kumar, Sadhanandham Shanmugasundaram, Ramesh Sankaran, Jayanty Venkata Balasubramaniyan, Vinod Kumar Balakrishnan, Jayanty S. N. Murthy, Thanikachalam Sadagopan, Nagendra Boopathy Senguttuvan

**Affiliations:** 1 Department of Cardiology, Sri Ramachandra Institute of Higher Education and Research (SRIHER), Chennai, Tamil Nadu, India; 2 Sri Ramachandra Institute of Higher Education and Research (SRIHER), Chennai, Tamil Nadu, India; 3 National Institute of Epidemiology, Indian Council of Medical Research (ICMR), Chennai, Tamil Nadu, India; 4 Section of Cardiology, Department of Internal Medicine, Max Rady College of Medicine, University of Manitoba, Manitoba, MB, Canada; 5 Department of Cardiology, Velammal Medical College Hospital and Research Centre, Madurai, India; 6 Department of Cardiology, McGill University Health Centre, Quebec, QC, Canada; 7 Department of Cardiology, Aster MIMS, Calicut, Kerala, India; 8 Bristol Heart Institute, Bristol, United Kingdom; 9 Department of Cardiology, Apollo Speciality Hospitals, Trichy, Tamil Nadu, India; 10 Department of Human Genetics, Sri Ramachandra Institute of Higher Education and Research (SRIHER), Chennai, Tamil Nadu, India

**Keywords:** cardiac MRI, coronary angiogram, coronary artery disease, delayed enhancement, heart failure, heart health, heart team, myocardial viability

## Abstract

**Aims:**

Our study aims to evaluate the influence of myocardial viability on therapeutic decision-making using cardiac MRI (CMR) and assess agreement and variability in heart-team therapeutic recommendations across different heart teams.

**Methodology:**

We included patients who underwent coronary angiography and CMR between January 2022 and January 2024 at a tertiary cardiac center. The revascularization strategy was independently determined by heart teams from four different centers, both before and after a review of CMR. The impact of CMR on therapeutic decisions was evaluated with a follow-up for long-term outcomes, while decision concordance before and after CMR was assessed using Cohen's kappa statistics. We calculated a novel residual viable syntax score by incorporating the myocardial viability into the residual syntax score.

**Results:**

Sixty-four patients were recruited. Indications for coronary angiography included ST elevation myocardial infarction (23, 36%), NSTEMI (15, 23.4%), unstable angina (7, 10.9%), and chronic coronary syndrome with limiting symptoms (19, 29.7%). Fifty-five (86%) patients had moderate to severe LV dysfunction. A proportion of 32.8% of the patients underwent revascularization in the study population in the form of coronary artery bypass grafting or percutaneous coronary intervention, while the rest were managed medically. Incorporation of CMR findings improved the consistency of treatment decisions across heart teams (Δ*κ* =  + 0.114, 95% confidence interval 0.01–0.22); however, these estimates should be interpreted cautiously because a total of 512 recommendations arose from repeated assessments of 64 patients and were not independent observations. Clinical-outcome analyses were exploratory because only 13 patients experienced major adverse cardiovascular outcomes during follow-up. The exploratory, non-validated residual viable SYNTAX score was not statistically significantly associated with clinical outcomes.

**Conclusion:**

CMR influenced heart-team revascularization recommendations and was associated with greater agreement in therapeutic decisions. These findings, including exploratory clinical-outcome analyses, require confirmation in larger studies that use cluster-aware statistical methods.

## Introduction

Cardiac MRI (CMR) is a highly useful tool for detailed, non-invasive assessment of heart structure, function, and tissue characterization, providing high spatial resolution and unrestricted imaging planes (1–3). Left ventricular (LV) dysfunction, both systolic and diastolic, is one of the important prognostic determinants of survival and quality of life in patients with coronary artery disease (CAD) (4, 5). Impaired resting LV systolic function was previously thought to be an irreversible process before the introduction and understanding of the concepts of stunned and hibernating myocardium (6). Hibernating myocardium is a viable, metabolically active, but chronically underperfused tissue with impaired contractility, which can recover with revascularization. In contrast, stunned myocardium is a transient dysfunction following an acute ischemic insult despite restored blood flow, which usually recovers spontaneously (6). In ischemic cardiomyopathy patients, identification of such hypocontractile but viable territory helps in decision-making regarding the benefit that results from revascularization (7). Revascularization improves oxygen delivery to the myocardium and restores aerobic metabolism.

Medical therapy for LV dysfunction predominantly includes renin–angiotensin–aldosterone system (RAAS) inhibitors, beta blockers, SGLT2 inhibitors, and mineralocorticoid receptor antagonists. Medical therapy, along with revascularization in the form of coronary artery bypass surgery (CABG) in ischemic cardiomyopathy patients, has been shown to improve survival compared with medical treatment alone (8). In the recent REVIVED-BCIS-2 (Revascularization for Ischemic Ventricular Dysfunction British Cardiovascular Intervention Society 2) trial, the role of revascularization in patients with severe LV dysfunction (EF < 35%) with extensive coronary artery disease despite their having a viable myocardium was observed to be questionable (9). However, 10-year follow-up data from the STITCH trial showed improved long-term survival in patients with severe LV dysfunction who underwent CABG compared with medical therapy alone (10). These findings highlight conflicting results in patients with LV dysfunction and a gray area in viability-guided revascularization. The chances of reversing LV dilatation-dysfunction and improving LV systolic function with medical therapy and/or revascularization are greater when the proportion of viable myocardium on non-invasive imaging is found to be significant (11, 12).

Cardiac MRI assesses myocardial viability by using the late gadolinium enhancement (LGE) technique with gadolinium-based contrast agents. These agents remain extracellular and help in characterizing viability based on differences in extracellular volume. As viable myocardium has less extracellular accumulation, it results in dark signals. However, in acute myocardial infarction (MI), these agents passively diffuse intracellularly through ruptured cell membranes and extracellularly in surrounding necrotic tissue, whereas in chronic infarcts, they concentrate in the collagenous scar that replaces necrotic tissue, resulting in brighter signals with LGE. Cardiac MRI can also identify the area at risk in the myocardium, which is the portion that is ischemic due to acute coronary occlusion and is at risk of infarction. The ideal result is revascularizing a viable myocardium, which can restore normal metabolism and improve contractile function (6). Delayed revascularization comes with the downside of no reflow phenomenon and reperfusion injury, while revascularizing a non-viable myocardium may not confer any benefit, while exposing the patient to procedural risks.

CMR has evolved into a gold-standard technique for assessing myocardial viability, while also providing information regarding global left ventricular function and regional wall motion abnormalities/viability. LV function is assessed using cine steady-state free precession sequences and a volumetric analysis of end systole and end diastole, while regional wall motion is studied using a standardized 17-segment model for systolic thickening and endocardial excursion. Other diagnostic modalities for assessment of myocardial viability include single-photon emission computed tomography (SPECT) and fluorine-18-fluorodeoxyglucose (^18^F-FDG) positron emission tomography (PET). SPECT assesses viability via active myocardial uptake and delayed redistribution, while-FDG PET is known for its superior sensitivity, image quality, and quantitative capabilities for myocardial blood flow (13). ACC and ESC guidelines currently have a class II recommendation for viability testing before revascularization for LV dysfunction (14, 15).

In this study, we aimed to (1) quantify agreement in heart team therapeutic decisions both before and after disclosure of CMR viability data in the overall cohort; (2) determine how CMR alters recommended management [CABG, percutaneous coronary intervention (PCI), or medical therapy]; (3) calculate anatomic complexity as viable SYNTAX and residual viable SYNTAX scores by subtracting non-viable territories to contextualize decisions; (4) evaluate outcomes [all-cause/CV death, HF hospitalization, non-fatal MI, composite major adverse cardiovascular outcomes (MACE)] and explore their relationships with baseline/viable/residual scores and the selected strategy.

## Materials and methods

We conducted a single-center observational study in the Department of Cardiology, Sri Ramachandra Institute of Higher Education and Research (SRIHER), Chennai, enrolling patients with coronary artery disease who underwent coronary angiography followed by CMR viability assessment between January 2022 and January 2024. Patients were identified using the institutional CMR database registry and Hospital Management Information System (HMIS). Baseline patient demographic data, clinical presentation, angiogram findings, and detailed cardiac MRI reports were deidentified and compiled for review to ensure patient confidentiality. Cardiac MRI was performed on a 3 Tesla scanner, and the following sequences were acquired: T1/T2 mapping, cine imaging, and LGE. Cardiac MRIs were reported by a single clinician, a cardiologist with specialized expertise and training in cardiac MRI. The institutional ethics committee approved the research study (IEC details: NI/21/APR/78/65).

*Patients and Public involvement*: Patients and/or the public were not involved in the design, conduct, reporting, or dissemination plans of this research.

*Methodology*: Each case was presented to four independent heart teams comprising an experienced interventional cardiologist and a cardiothoracic surgeon within each team. The teams were labeled as A, B, C, and D, respectively. No experts from SRIHER were included on the review panels to avoid institutional bias. A web-based platform was used to present each case to the expert teams individually in two parts:

1. Coronary angiograms of each patient, supported by clinical data, were provided initially to each team individually. They were also provided with the SYNTAX score for assessing anatomic severity. Later, they were asked to recommend a treatment strategy with the available information. (C = CABG; P = PCI; M = medical management) and their decisions were noted down.
2. Following that, CMR viability data were disclosed, and each team was asked to reassess and provide their treatment strategy, and their decision was again noted down.

Importantly, all the teams were blinded to the actual treatment of the patients.

Following cardiac MRI, the SYNTAX score was recalculated for all the patients based on viable territory as the viable SYNTAX score.

Viable SYNTAX score = Baseline SYNTAX Score – Non-viable vessel (LAD/LCX/RCA) SYNTAX score. (Non-viable SYNTAX is calculated from the vessel supplying segments with transmural LGE enhancement)

Baseline SYNTAX score = viable SYNTAX score + SYNTAX score of non-viable territory

We also calculated a novel residual viable SYNTAX score, which we defined as the SYNTAX score of non-revascularized but viable territory for the particular patient.

Residual viable SYNTAX score = residual SYNTAX score – non-viable territory(ies) SYNTAX score

Patients were followed up for MACE, including all-cause mortality, cardiovascular mortality, rehospitalization due to non-fatal myocardial infarction, and heart failure.

### Study outcomes

*Primary Outcome*: The primary outcome of the study was the agreement between the heart team's therapeutic decisions both before and after CMR in the overall group.

*Secondary Outcomes*: The secondary outcomes of the study included the following:

1. *Effect of CMR on therapeutic decisions:* Change in the recommended management (e.g., CABG, PCI, or optimal medical therapy) after incorporation of CMR viability findings in the overall cohort.
2. *Intrateam decision agreement*: Concordance within the same heart team both before and after reviewing CMR data, to quantify the influence of CMR on team-specific decision-making.
3. *Interteam consensus*: Consistency of recommended strategies across different heart teams both before and after CMR, assessing whether CMR enhances consensus for patients with CAD and LV dysfunction.
4. *Clinical outcomes (exploratory)*: Events including all-cause death, non-fatal myocardial infarction, repeat revascularization, heart-failure readmission, stroke, and composite endpoints (e.g., MACE as CV death, non-fatal MI, repeat revascularization, HF admission, and stroke).
5. *Residual viable SYNTAX (rv SYNTAX) and outcomes (exploratory)*: Association between the residual viable SYNTAX score and subsequent clinical outcomes.

### Sample size calculation

The sample size was determined to ensure adequate precision in estimating interrater agreement for therapeutic recommendations using Cohen's kappa. We targeted an expected kappa of 0.40 with a two-sided 90% confidence interval (CI) of 0.20. Assuming an expected proportion of approximately 0.35 per therapeutic recommendation, the study included 64 patient cases. Each case was assessed repeatedly by four heart teams at two time points (pre-CMR and post-CMR), yielding 512 therapeutic recommendations (256 pre-CMR and 256 post-CMR). These recommendations represented repeated, clustered assessments within the same 64 patients and were therefore not independent observations. This calculation was based on the criterion:

$$n\;\geq \frac{Z_{1\text{-}a\;/\;2}^{2}}{d^{2}}$$

where *z*_1–*a*/2_ = 1.645 for a 90% CI, *d* = 0.20 is the desired margin of error, and *Q* is a function of the expected kappa and marginal proportions.

In the study design, four multidisciplinary heart teams provided therapeutic recommendations for 64 patients at two time points (pre-MRI and post-MRI), yielding a total of 512 recommendations (256 pre-MRI and 256 post-MRI). This design permitted descriptive and exploratory assessments of agreement across scenarios, teams, and therapeutic strategies. Because the recommendations were clustered within patients, the number of recommendations should not be interpreted as 512 independent observations.

### Statistical analysis

Data were entered into Microsoft Excel. Continuous variables were expressed as mean ± standard deviation or median (interquartile range), based on the normality of the distribution. Categorical variables were expressed as percentages. Percentages (along with 95% CI) of therapeutic decisions pre- and post-Cardiac Magnetic Resonance Imaging (CMRI) were compared within rating teams and overall. Levels of agreement between therapeutic decisions made pre- and postcardiac MRI were assessed using Cohen's kappa (with 95% CI) statistics for each treatment category, CABG (C), PCI (P), and medical management (M), as well as overall agreement across all categories. Because multiple recommendations were obtained for each patient, the kappa estimates and their confidence intervals were interpreted cautiously as descriptive measures of agreement; the repeated assessments were not treated as fully independent observations. We performed a multiple logistic regression analysis for MACE using various clinical and angiographic parameters, and Left Ventricular Ejection Fraction (LVEF). Given the limited number of MACE events, all analyses of clinical outcomes, including associations with MACE, were exploratory. A two-tailed *p*-value of <0.05 was considered statistically significant. SPSS Version 28 (IBM, USA) and R (R 4.5.1 binary for macOS, Denmark) were used for statistical analysis.

## Results

The median (IQR) age of the study population was 60 (14) years, with 80% of the patient population aged above 50 years. A proportion of 17.2% of the study population was female. The major cardiovascular risk factors noted in the study population were hypertension (46.9%) and type 2 diabetes mellitus (65.6%). The most common presentation was delayed (or out-of-window period) ST elevation myocardial infarction (STEMI) (36%), followed by chronic coronary syndrome (CCS) with limiting symptoms (29.7%) and non-STEMI (23.4%). A proportion of 15.6% of patients had acute decompensated heart failure on presentation, and 24 (37.5%) patients had severe LV dysfunction at baseline. Patient demographics and other details are provided in Table 1.

**Table 1:** Characteristics of the study participants.

Patient characteristics	Number of patients (%)/ median (IQR) of total cases
Age (years) <40 40–50 50–60 60–70 >70 Median age (IQR) Male sex Female sex	3 (4.7) 10 (15.6) 21 (32.8) 17 (26.6) 13 (20.3) 60 (14.5) 53 (82.8%) 11 (17.2%)
T2DM Yes	42 (65.6%)
HTN Yes	30 (46.9%)
LV Function Mild LV dysfunction (40%–50%) moderate LV Dysfunction (30%–40%) severe LV Dysfunction (<30%)	9 (14%) 31 (48.4%) 24 (37.5%)
Diagnosis ACS-AWMI ACS-IWMI ACS-NSTEMI CCS (class II symptoms) CCS (>class II symptoms) USA	20 (31.3%) 3 (4.7%) 15 (23.4%) 9 (14.1%) 10 (15.6%) 7 (10.9%)
Intervention CABG PCI ICD	11 (17.2%) 10 (15.6%) 2 (3.1%)

A CMR analysis revealed the left anterior descending (LAD) territory to be the major non-viable territory in the study population (32.50% of cases), the left circumflex (LCx) was non-viable in 15.6% of cases, and the right coronary artery (RCA) was non-viable in 23.4% of cases. The median (IQR) baseline SYNTAX score of all patients was 24.25 (15), whereas the median (IQR) viable SYNTAX score calculated from subtracting the non-viable vessel SYNTAX score from the baseline SYNTAX score was 10 (14), indicating that the disease is less complex for revascularization when cardiac MRI suggested that viability is accounted for. CMR data are presented in Table 2. Eleven patients (17.18%) in the study population underwent CABG, 10 patients (15.6%) underwent a percutaneous coronary intervention (seven patients—single-vessel PCI, three patients—two-vessel PCI), while the remaining patients were managed medically. Two patients also underwent device therapy in the form of an implantable cardioverter defibrillator (ICD). We also calculated a novel, exploratory, non-validated residual viable SYNTAX score, which showed a median (IQR) of 3.5 (12).

**Table 2:** MRI characteristics of the study population.

MRI characteristics	*N* (%)/median (IQR) of total cases
LAD Not viable Viable Mixed	32 (50) 21 (32.8) 11 (17.2)
LCX Not viable Viable Mixed	10 (15.6) 51 (79.7) 3 (4.7)
RCA Not viable Viable Mixed	15 (23.4) 43 (67.2) 6 (9.4)
LVEF (%)	27.50 (20)
RVEF (%)	50 (21.25)
LVEDV (mL/m^2^)	85 (30)
LVESV (mL/m^2^)	60 (21)
LV cardiac index (L/min/m^2^)	2 (0.44)
RVEDV (mL/m^2^)	54 (20)
RVESV (mL/m^2^)	27 (15)
RV cardiac index (L/min/m^2^)	2 (0.36)

EF, ejection fraction; EDV, end-diastolic volume; ESV, end-systolic volume, RV, Right ventricle, LV, Left ventricle.

### Outcome analysis

We analyzed 512 therapeutic recommendations (256 pre-CMR and 256 post-CMR) from four multidisciplinary heart teams (64 decisions per team at each time point), covering coronary revascularization strategies: CABG (C), PCI (P), or medical therapy (M). These recommendations were clustered within patients and should not be interpreted as independent observations (Table 3). Of the 70 therapeutic decisions initially designated for CABG, 48 (69%, 95% CI 58–79) remained CABG after CMR, 15 (21%, 95% CI 12–31) switched to PCI, and seven (10%, 95% CI 3–17) shifted to medical therapy. Among 88 decisions initially planned for PCI, 69 (78%, 95% CI 70–87) remained PCI, and 17 (19%, 95% CI 11–28) were reassigned to medical therapy. Of the 98 decisions initially recommended for medical therapy, 79% (95% CI 70–87) continued with medical therapy, 15% (95% CI 8–22) shifted to PCI, and 6% (95% CI 1–11) switched to CABG (Table 3).

**Table 3:** Percentage of therapeutic decisions both pre- and postcardiac MRI by individual heart teams and overall.

		Team A (postcardiac MRI)			Total
		C (%) [95% CI]	P (%) [95% CI]	M (%) [95% CI]	
Team A (precardiac MRI)	C	12 (63) [38, 84]	7 (37) [16, 62]	0 (0)	19
	P	1 (4) [0, 17]	18 (75) [53, 90]	5 (21) [71,42]	24
	M	2 (10) [1, 30]	2 (10) [1, 30]	17 (80) [58, 95]	21
Total		15	27	22	64

		Team B (postcardiac MRI)			Total
		C (%) [95% CI]	P (%) [95% CI]	M (%) [95% CI]	
Team B (precardiac MRI)	C	20 (91) [70, 99]	1 (5) [0,23]	1 (5) [0, 23]	22
	P	1 (6) [0, 30]	10 (63) [35, 85]	5 (31) [11, 59]	16
	M	0 (0)	2 (8) [0, 25]	24 (92) [75, 99]	26
Total		21	13	30	64

		TEAM C (postcardiac MRI)			Total
		C (%) [95% CI]	*P* (%) [95% CI]	M (%) [95% CI]	
Team C (precardiac MRI)	C	9 (69) [39, 91]	2 (15) [2, 45]	2 (15) [2, 45]	13
	P	0 (0)	18 (95) [74, 100]	1 (5) [0, 26]	19
	M	4 (13) [4, 29]	7 (22) [9, 40]	21 (66) [47, 81]	32
Total		13	27	24	64

		TEAM D (postcardiac MRI)			Total
		C (%) [95% CI]	*P* (%) [95% CI]	M (%) [95% CI]	
Team D (precardiac MRI)	C	7 (44) [19, 70]	5 (31) [11, 52]	4 (25) [7, 52]	16
	P	0 (0)	23 (79) [60, 92]	6 (21) [8, 40]	29
	M	0 (0)	4 (21) [6, 46]	15 (79) [54, 94]	19
Total		7	32	25	64

		Postcardiac MRI			Total
		C (%) [95% CI]	*P* (%) [95% CI]	M (%) [95% CI]	
Precardiac MRI	C	48 (69) [56, 79]	15 (21) [13, 33]	7 (10) [4, 20]	70
	P	2 (2) [0, 7]	69 (78) [68, 87]	17 (19) [12, 29]	88
	M	6 (6) [2, 13]	15 (15) [9, 24]	77 (79) [69, 86]	98
Total		56	99	101	256

C, CABG; P, PCI; M, medical management.

### Primary outcome

The primary outcome was overall agreement between the heart team's therapeutic decisions both before and after CMR. We quantified this by Cohen's *κ* for each category—CABG (C), PCI (P), and medical therapy (M)—and overall. Overall agreement improved after CMR, driven mainly by gains in CABG and medical-therapy categories (Table 4).

- CABG: *κ* increased from 0.305 (95% CI 0.20–0.40) pre-CMR to 0.451 (0.35–0.55) post-CMR; Δ*κ* =  + 0.146 (95% CI 0.01–0.29), suggesting a descriptively greater agreement after CMR.
- PCI: *κ* rose slightly from 0.261 (0.16–0.36) to 0.292 (0.19–0.39); Δ*κ* =  + 0.031 (95% CI −0.11–0.17), suggesting minimal change.
- Medical therapy: *κ* improved from 0.350 (0.25–0.45) to 0.526 (0.42–0.62); Δ*κ* =  + 0.176 (95% CI 0.04–0.32), a descriptively higher level of agreement.

**Table 4:** Agreement of therapeutic decisions both pre- and postcardiac MRI by individual categories of decision.

Categories	Precardiac MRI		Postcardiac MRI		Change in kappa (post–pre) (95% CI)
	Kappa	95% CI	Kappa	95% CI	
C	0.305	(0.20, 0.40)	0.451	(0.35, 0.55)	0.146 (0.01, 0.29)
P	0.261	(0.16, 0.36)	0.292	(0.19, 0.39)	0.031 (−0.11, 0.17)
M	0.350	(0.25, 0.45)	0.526	(0.42, 0.62)	0.176 (0.04, 0.32)
Overall	0.306	(0.23, 0.37)	0.420	(0.34, 0.49)	0.114 (0.01, 0.22)

C, CABG; P, PCI; M, medical management.

### Other outcomes

**Figure 1:**
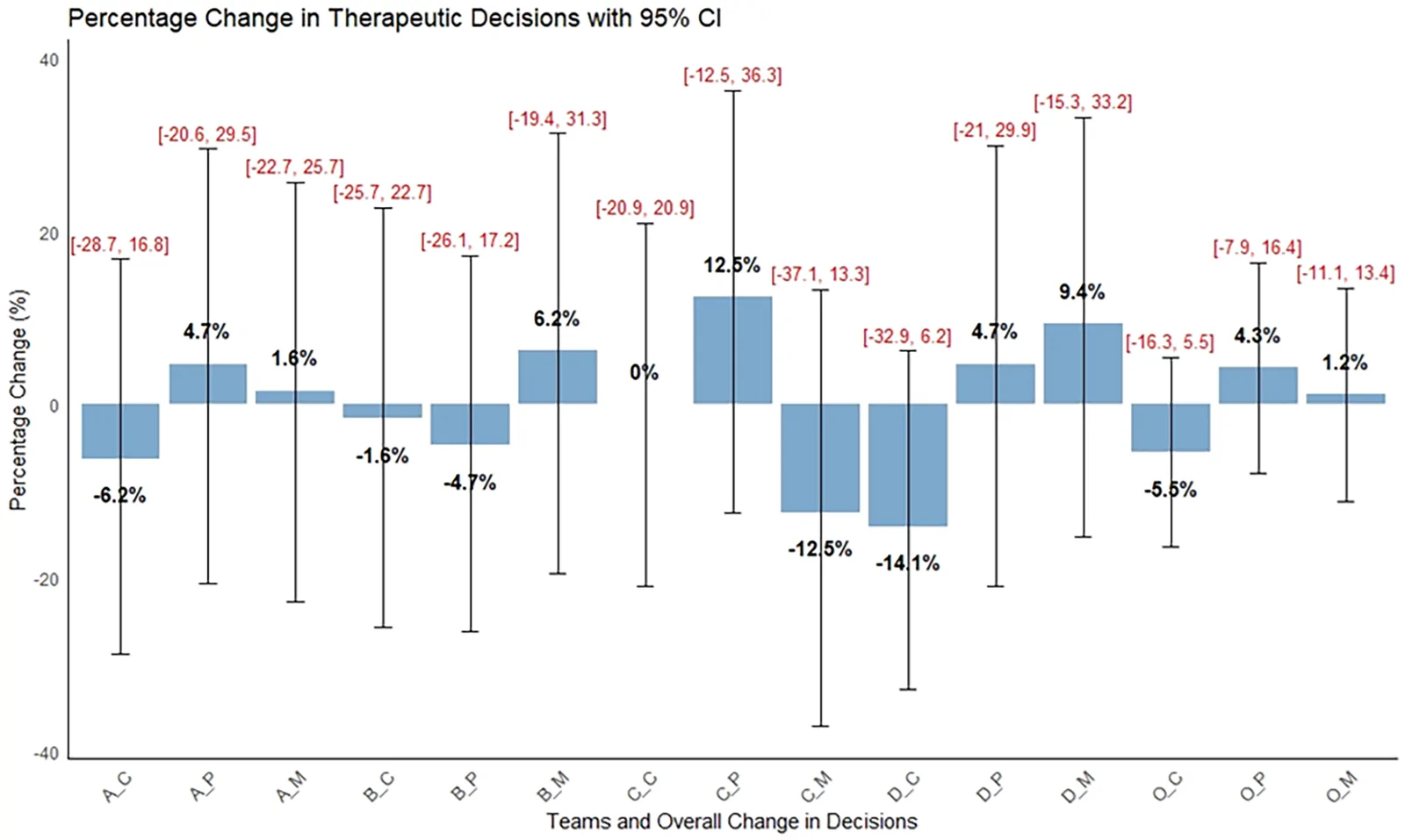
Percentage changes in therapeutic decisions with 95% CI (A, B, C, and D teams and O, overall; C, CABG; P, PCI; M, medical management).

Cardiac MRI substantially influenced and changed the therapeutic decision-making across all teams. Overall, 31% of decisions initially considered for CABG or PCI experienced a change in management post-MRI, with a significant number shifting toward less invasive strategies. Medical management plans showed the highest consistency (79%), while CABG decisions were revised most frequently. These findings highlight the pivotal role of cardiac MRI in optimizing and individualizing coronary revascularization strategies (Figure 1).

### Individual team decision outcomes

Team A

- CABG (*n* = 19): 12 remained CABG (63%, 95% CI 38–84); 7 (37%, 95% CI 16–62) shifted to PCI.
- PCI (*n* = 24): 18 remained PCI (75%, 95% CI 53–90); 5 (21%, 95% CI 7–42) were reclassified to medical therapy.
- Medical therapy (*n* = 21): 17 remained medical therapy (80%, 95% CI 58–95); 20% moved to revascularization.

Team B

- CABG (*n* = 22): 20 remained CABG (91%, 95% CI 70–99); 5% shifted to PCI and 5% to medical therapy.
- PCI (*n* = 16): 10 remained PCI (63%, 95% CI 35–85); five (31%, 95% CI 11–59) shifted to medical therapy.
- Medical therapy (*n* = 26): 24 remained medical therapy (92%, 95% CI 75–99).

Team C

- CABG (*n* = 13): nine remained CABG (69%, 95% CI 39–91); 15% shifted to PCI and 15% to medical therapy.
- PCI (*n* = 19): 18 remained PCI (95%, 95% CI 74–100); 5% moved to medical therapy.
- Medical therapy (*n* = 32): 21 remained medical (66%, 95% CI 47–81).

Team D

- CABG (*n* = 16): seven remained CABG (44%, 95% CI 19–70); 31% shifted to PCI and 25% to medical therapy.
- PCI (*n* = 29): 23 remained PCI (79%, 95% CI 60–92); six (21%, 95% CI 8–40) shifted to medical therapy.
- Medical therapy (*n* = 19): 15 remained medical (79%, 95% CI 54–94); 21% moved to PCI, indicating a notable CMR-driven shift toward revascularization.

CABG recommendations showed the greatest variability in Team D (only 44% remained CABG post-CMR).

### Clinical outcomes

**Figure 2:**
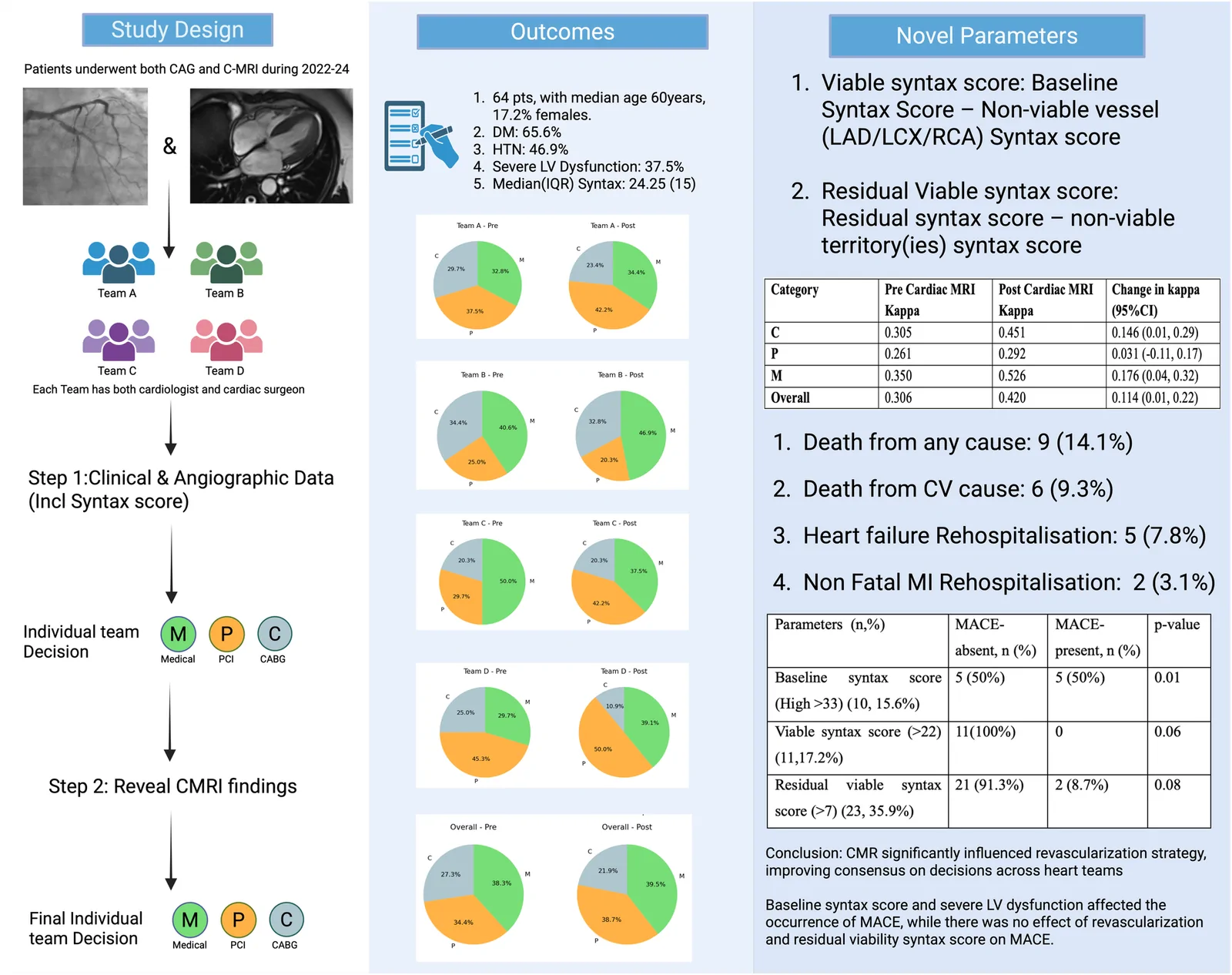
Graphical abstract of the study.

At a median (IQR) follow-up of 14 months (9 months), six patients (9.3%) died from cardiovascular causes, five (7.8%) patients were rehospitalized for heart failure, and two (3.1%) were rehospitalized for non-fatal myocardial infarction (Table 5). A total of 13 patients (20.3%) experienced MACE events on follow-up. A score of residual SYNTAX of more than 7 has been shown to impact clinical outcomes. Hence, an rv (residual viable) SYNTAX of seven or more was considered significant. Additional parameters analyzed were baseline SYNTAX, diabetes mellitus, hypertension, chronic kidney disease, viable SYNTAX score>22, residual viable SYNTAX score>7, severe LV dysfunction, or having done any revascularization (PCI and CABG together). In these exploratory analyses, a higher baseline SYNTAX score and severe LV dysfunction were statistically associated with MACE. No statistically significant association with MACE was detected for diabetes mellitus, hypertension, chronic kidney disease, viable SYNTAX score, residual viable SYNTAX score >7, or revascularization (Table 6). The incorporation of myocardial viability into residual anatomical disease burden, reflected by the rv SYNTAX score, was not statistically significantly associated with clinical outcomes in this exploratory analysis (Figure 2).

**Table 5:** Clinical outcomes.

Patient-related clinical outcomes	Number of patients (%)
Composite outcome of death from any cause	9 (14.1)
Death from cardiovascular cause	6 (9.3)
Rehospitalization for heart failure	5 (7.8)
Rehospitalization for non-fatal myocardial infarction	2 (3.1)

**Table 6:** Correlation of major adverse cardiovascular events with different parameters.

Parameters (*n*, %)	MACE-absent (*n*, %)	MACE-present (*n*, %)	*p*-Value
Female sex [11, (17.2%)]	10, 90.9	1, 9.1	0.31
Hypertension [30, (46.9%)]	24, 80	6, 20	0.95
Diabetes mellitus (43, 67.2%)	36, 83.7	7, 16.3	0.25
Chronic kidney disease (2, 3.1%)	1, 50	1, 50	0.29
Baseline syntax score (High>33)(10, 15.6%)	5, 50	5, 50	0.01
Viable syntax score (score>22)(11, 17.2%)	11, 100	0	0.06
Residual viable syntax score (more than 7) 23, 35.9%)	21, 91.3	2, 8.7	0.08
Severe LV dysfunction (24, 37.5%)	15, 62.5	9, 37.5	0.008
Revascularization (21, 32.8%)	17, 81	4, 19	0.86

## Discussion

Interteam agreement on revascularization strategy improved after CMR, with the greatest gains in the CABG and medical therapy categories. Approximately one-third of patients had their planned treatment modality changed post-CMR.A significant shift was observed toward less invasive treatment strategies. A higher baseline SYNTAX score and severe LV dysfunction were associated with MACE, whereas no statistically significant association between revascularization and MACE was detected in this exploratory analysis, rather than implying evidence of absence of a treatment effect. The newly proposed rv SYNTAX score showed no significant relationship with MACE.

Cardiac MRI provides high-resolution images without ionizing radiation and hence is considered to be superior to nuclear imaging techniques such as SPECT and PET (16). The high spatial resolution and direct tissue characterization help in better quantification of scar burden, which is a key component of viability assessment and can be done in a single examination. Newer nuclear imaging modalities like D-SPECT, which are cardiac–specific, has shown comparable efficacy to CMR (17). The choice of imaging modality in different centers can also be influenced by the availability of local resources and expertise.

**Figure 3:**
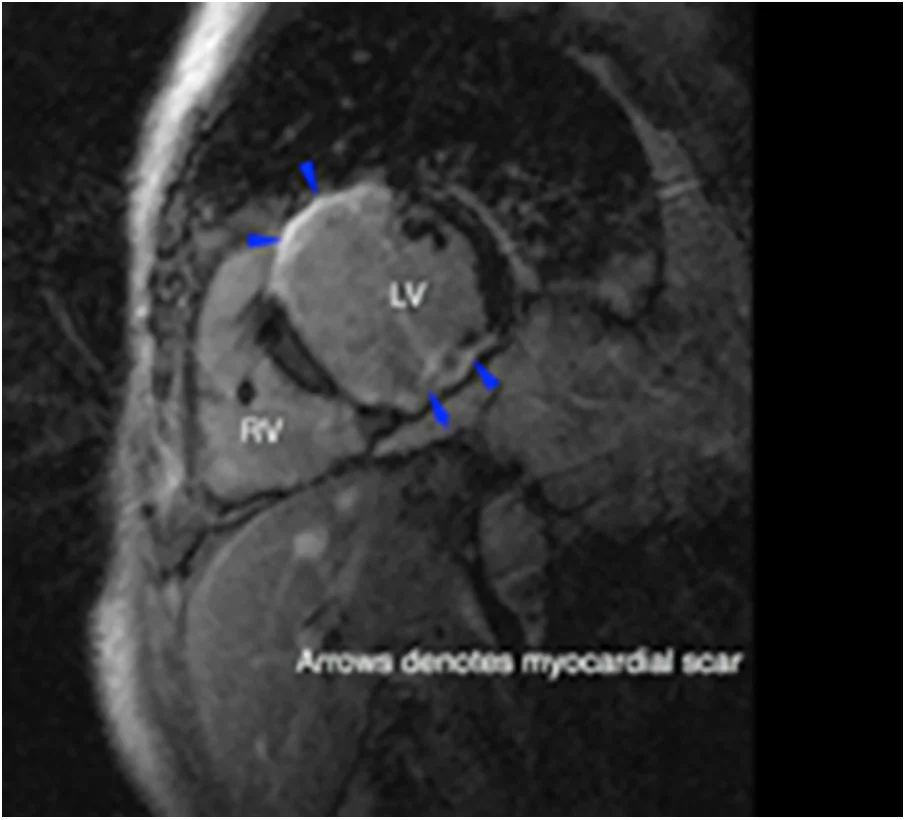
A CMR image showing myocardial scarring.

**Figure 4:**
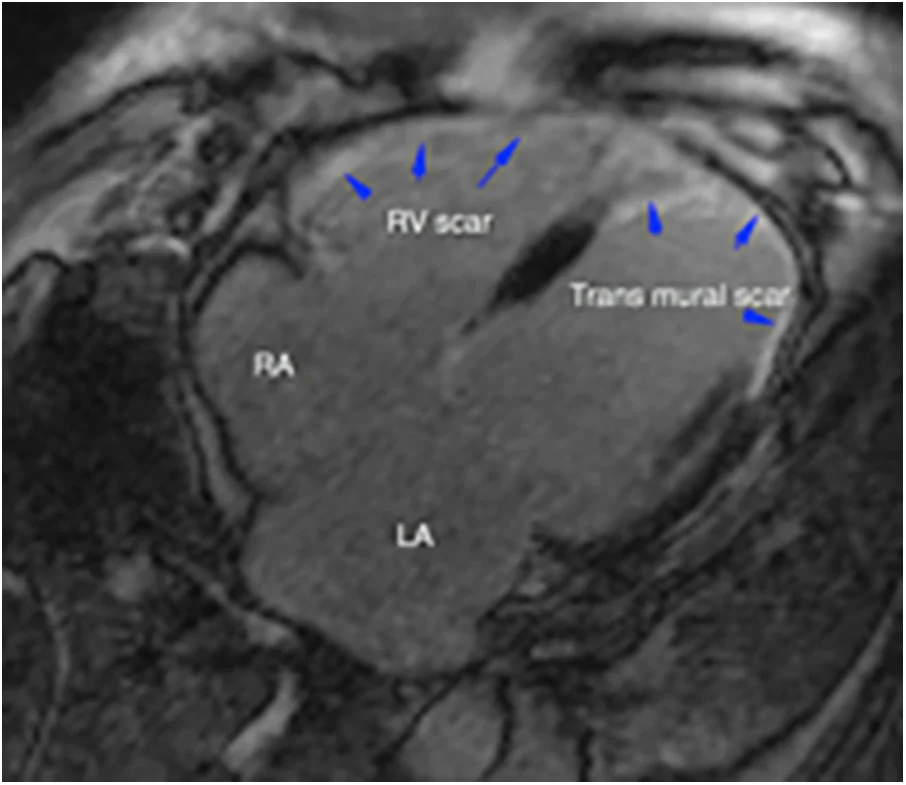
A CMR image showing a transmural scar in the LV and an RV scar.

**Figure 5:**
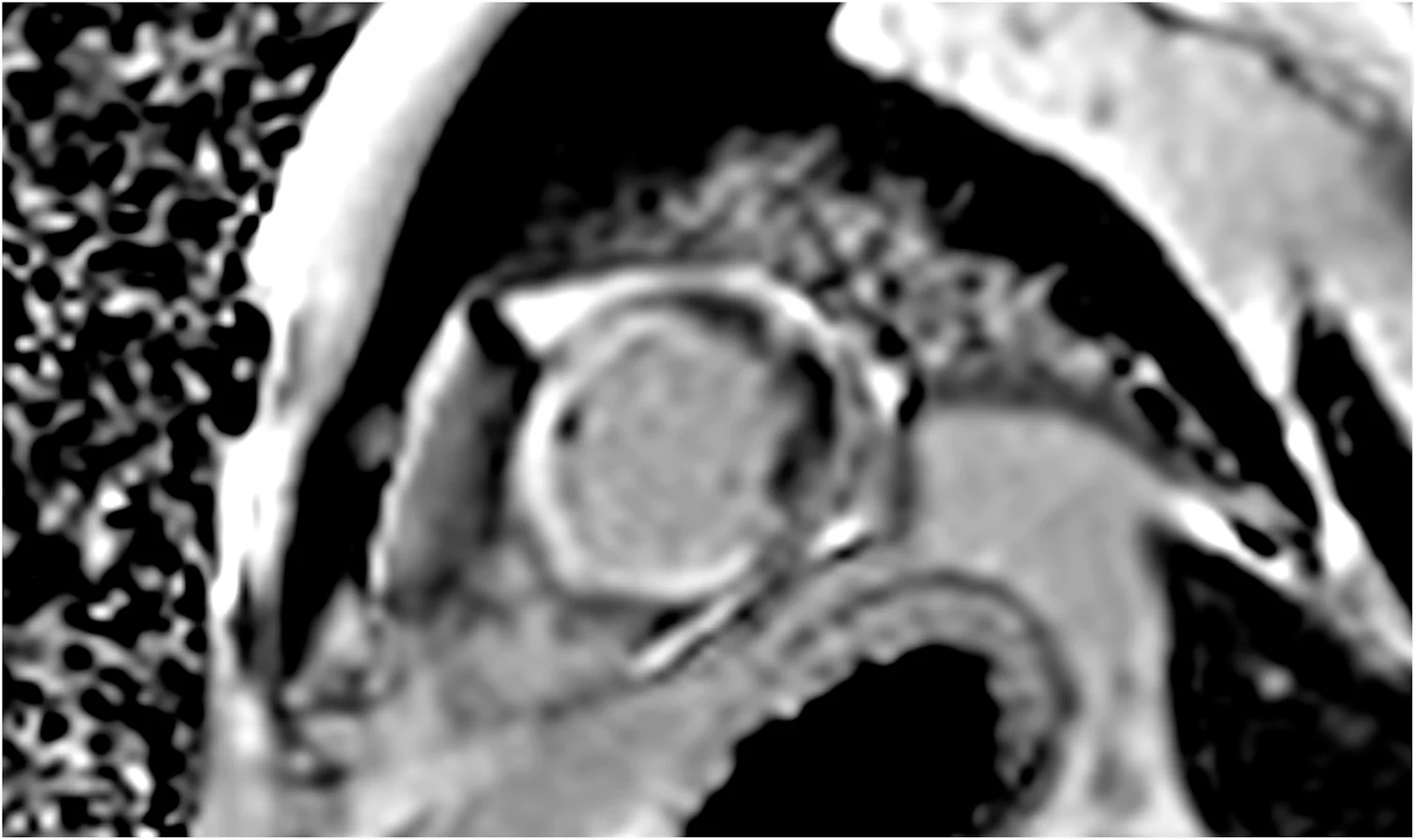
CMR at the short-axis apical level demonstrating LGE.

**Figure 6:**
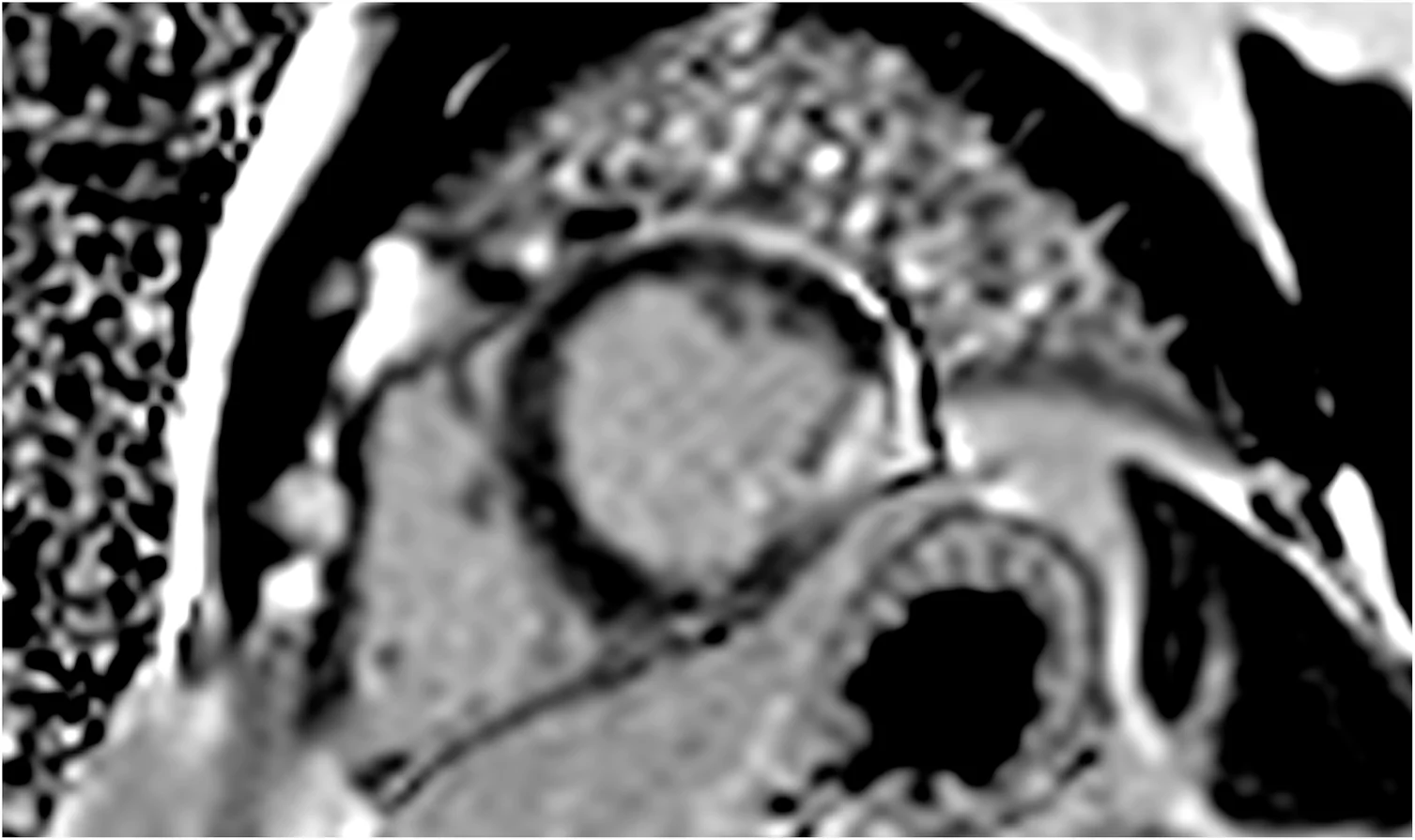
CMR at the short-axis midventricular level showing LGE.

**Figure 7:**
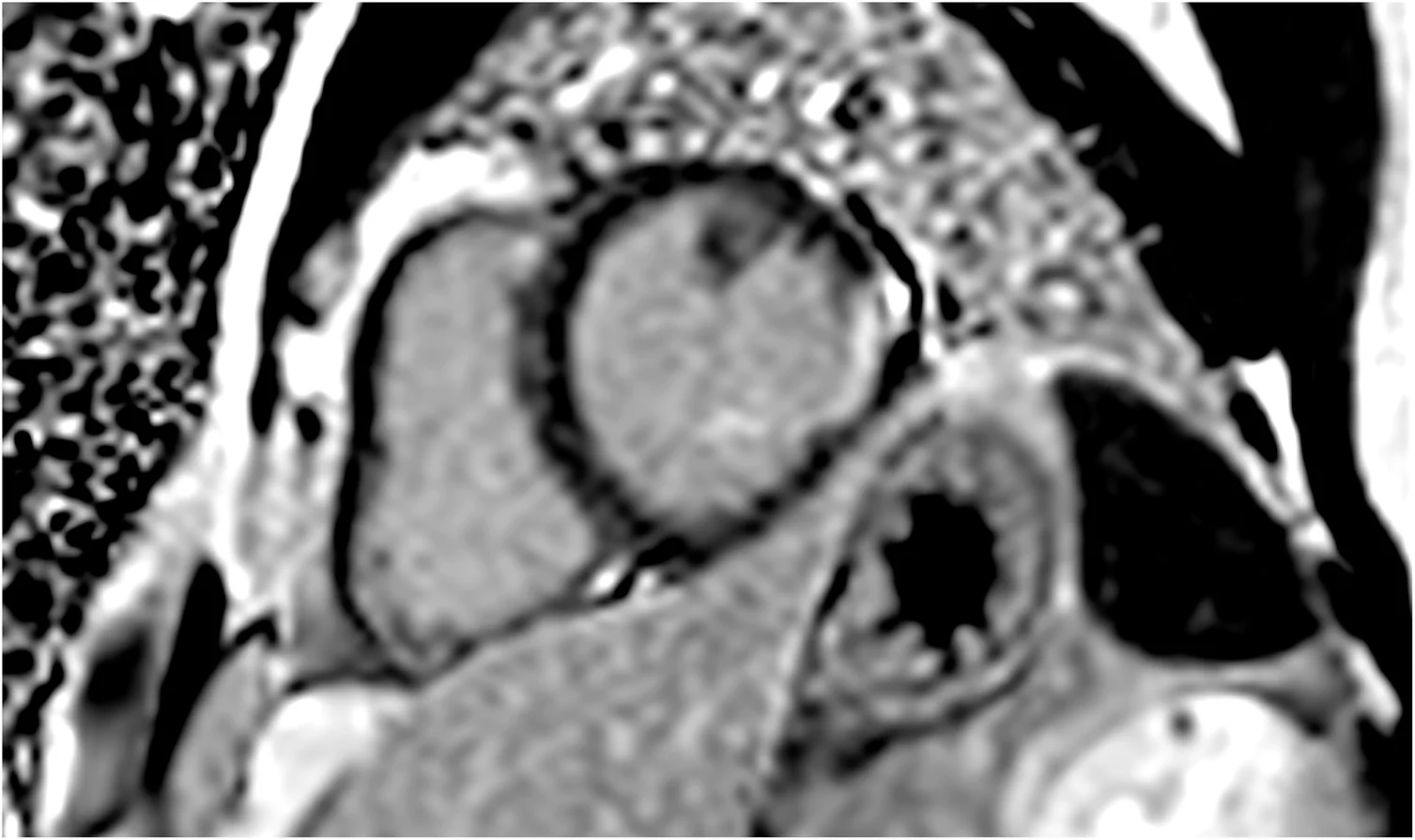
CMR at the short-axis basal level showing LGE.

Non-viable myocardium refers to the myocardial segments that have lost contractile function and are unlikely to recover even with revascularization. After infarction or chronic ischemia, the myocytes in these segments undergo necrosis and are replaced by collagen-rich fibrotic tissue (replacement fibrosis), which eventually leads to the formation of a scar. The differentiation between viable and non-viable segments is made by the LGE technique of CMR. LGE was assessed using the standardized 17 segment model, each segment graded for the extent of transmural enhancement. Segments with more than 50% transmural enhancement with gadolinium are considered to be non-viable (18) (Figures 3–7). A meta-analysis done by the Medical Advisory Secretariat of Ontario across 11 studies between the period of 2005 and 2009 showed a pooled sensitivity and specificity of 84.5% and 71%, respectively, for predicting regional functional recovery (19). Whether to revascularize patients with CAD and LV dysfunction or to identify the appropriate type of revascularization in such patients is a matter of debate. Although the initial 1-year result of the STICH (Surgical Treatment for Ischemic Heart Failure) study showed no benefit of revascularization, the long-term 10-year follow-up data showed improved survival in the CABG arm as compared to the medical therapy arm (10). However, assessment of myocardial viability did not predict which patients would benefit from surgical revascularization, as patients with or without viability had similar responses to CABG. In contrast to STICH, the REVIVED-BCIS-2 (9) study showed no benefit of revascularization for patients who underwent viability-based revascularization as compared to medical therapy alone. In addition to the hard endpoint, it also showed no change in the angina score or QoL and no improvement in LV ejection fraction, which contradicts our clinical observations.

In our study, the agreement between therapeutic decision-making both before and after cardiac MRI was assessed using Cohen's kappa statistics. For decisions involving CABG, the kappa improved from 0.305 to 0.451 post-CMR, and improvement was noted from 0.350 to 0.526 for decisions involving medical management. CMR had a limited impact on the PCI consensus both pre- and post-MRI. Previous studies also highlighted the role of CMR and other viability imaging modalities in refining treatment plans for patients with coronary artery disease and LV dysfunction. Early observational data, such as the meta-analysis by Allman et al., strongly suggested a survival benefit for revascularization in patients with a viable myocardium, forming the basis of the debate around viability assessment (20). However, subsequent studies such as the HEART trial (which was underpowered and terminated early) and the PAAR 2 trial, which used FDG-PET for viability assessment, failed to show a consistent overall benefit of viability-guided revascularization (21). Hence, the clinical significance of viability assessment was always a matter of debate, with conflicting results reported from earlier studies. The Euro CMR registry was a multicenter registry with consecutive enrollment of patients from 57 centers across 15 countries in Europe, involving more than 27,000 patients, in which 14.7% of the patients underwent CMR for viability assessment. The registry evaluated the indications, image quality, safety, and impact on patient management by CMR and the prognostic potential of CMR. The registry showed that CMR impacted decision-making and management in nearly two-thirds of patients (61.8%) either by providing a new diagnosis or/and resulting in therapeutic consequences (22).

Abbasi et al. studied 150 consecutive patients with heart failure and an ejection fraction ≤50% referred for CMR. They found that CMR had a significant clinical impact in 65% of patients, leading to changes in medication, revascularization strategies, and device therapy or a completely new diagnosis in 30% of patients (23). Unlike the Euro CMR registry or the study by Abbasi et al., which examined the impact of cardiac MRI on both ischemic and non-ischemic cases, our study focused on the influence of CMR on therapeutic decision-making in patients with coronary artery disease alone. Also, unlike these studies, CMR data were interpreted by a single reader, eliminating real-world inter-reader variability. However, the involvement of multiple heart teams in our study allows for better consensus and reduces the possibility of individual bias. A similar trial design to our study was used in the SYNTAX III Revolution trial (24), where the uniformity of decisions from Fractional Flow Reserve (FFR) derived from CT coronary angiography and invasive angiography was studied across different heart teams.

We evaluated the impact of CMR on clinical decision-making across four multidisciplinary heart teams. Among cases initially designated for CABG, 69% remained CABG after CMR, while 21% were reclassified to PCI and 10% to medical therapy, likely reflecting CMR-identified patterns of a viable/non-viable myocardium and lower effective disease complexity. This underscores the value of viability assessment before surgical revascularization in CAD (25). Among cases initially planned for PCI, 20% were deemed less suitable for intervention after CMR, consistent with non-viable territories. Conversely, 21% of patients initially assigned to medical therapy were shifted to revascularization based on CMR findings. Overall, CABG recommendations decreased, whereas those of PCI and medical therapy increased—suggesting a more precise alignment of treatment with viability burden and procedural risk (26). Notably, the viable SYNTAX score (accounting for non-viable territories) was lower than the baseline SYNTAX score, favoring targeted revascularization of viable myocardium and contributing to shifts from CABG to PCI.

The clinical outcomes and prognosis of the study population were similar to those of the national heart failure registry data (27). Compared with the National Heart Failure Registry of India (27) or the Revived BCIS 2 study (9), we found a lower CV mortality rate. Possible reasons are a younger population, with all patients undergoing viability-guided revascularization in the appropriate persons based on CMR, institutional follow-up with strict adherence to guideline-directed medical therapy, and proper vaccination protocol. However, the low number of total MACE events, amounting to fewer events per variable, can make this finding hypothesis-generating and exploratory in nature.

**Figure 8:**
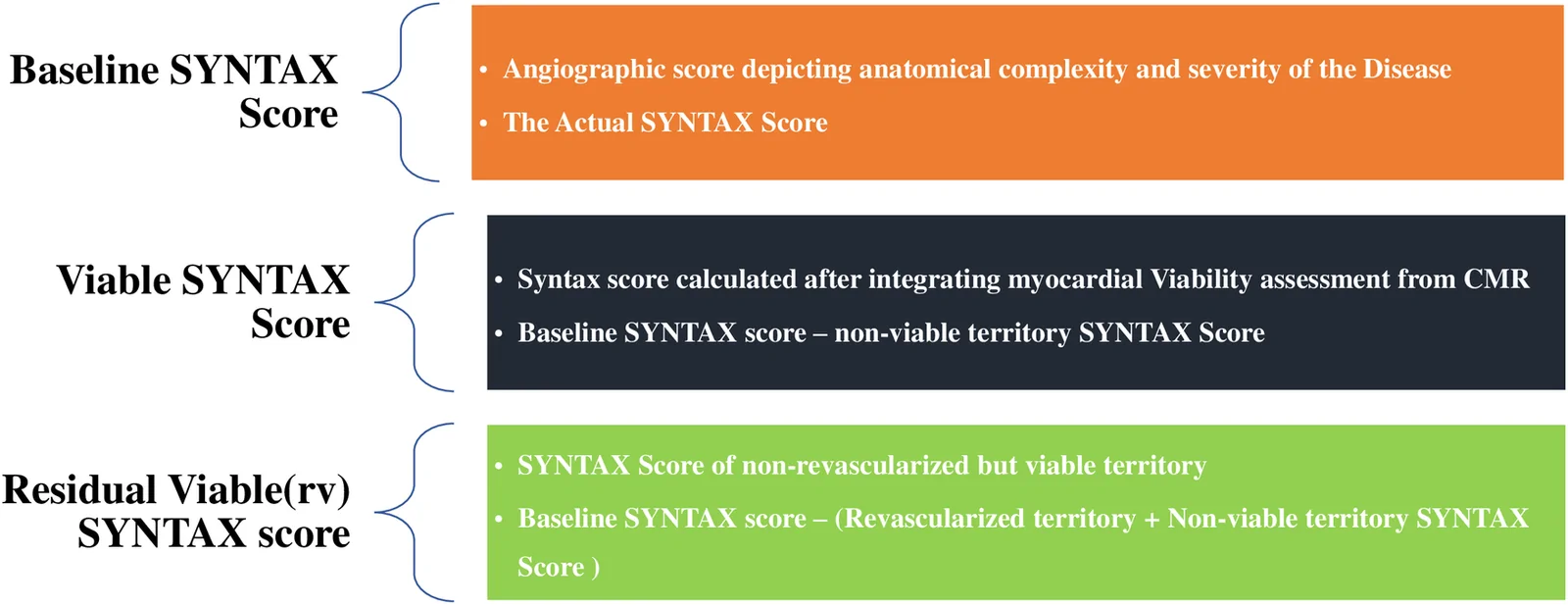
Various syntax score definitions.

We derived a residual viable SYNTAX (rv SYNTAX) score to examine associations with clinical outcomes. Unlike the traditional residual SYNTAX score described (28), rv SYNTAX explicitly incorporates the viability of non-revascularized myocardial territories, integrating anatomic complexity with tissue viability. In our cohort, a higher baseline SYNTAX score and severe LV dysfunction were significantly associated with MACE, whereas rv SYNTAX was not (Figure 8). Although the rv-SYNTAX score was not significantly associated with clinical outcomes, this finding may be attributable to the modest sample size and short follow-up time. Moreover, viability-guided revascularization was also not associated with a subsequent reduction in MACE, likely reflecting the relatively short follow-up duration, which may have been insufficient to capture its long-term clinical benefits. Therefore, these results may be considered hypothesis-generating and should be confirmed in larger prospective studies with longer follow-up times.

### Limitations

The first limitation is that the cohort was modest and the follow-up relatively short, limiting the power to detect differences in clinical endpoints. Nevertheless, each case was independently reviewed by four heart teams, yielding 512 discrete recommendations (256 pre-CMR and 256 post-CMR), thereby increasing the number of decision observations. Also, the modest representation of females in the cohort (17.2%) limits the generalizability of the findings.

Second, all CMR studies were interpreted by a single clinician, which introduces potential reader bias and eliminates inter-reader variability, which are relevant to real-world applicability. Also, the heart teams were not provided with information such as segment-level LGE data, which could have influenced changes in management strategy. Future work should employ multireader adjudication (or a core laboratory), longer follow-up times, and larger multicenter cohorts.

Also, the clustered, non-independent structure of the data may lead to underestimated confidence intervals around Cohen's kappa estimates. However, as this clustering is identical in both pre- and post-CMR conditions, the directional inference about improvement in consensus (Δ*κ* > 0) is less susceptible to clustering bias than the absolute kappa values themselves. Hence, future larger studies should employ cluster-adjusted or mixed-effects agreement models for a more robust inference.

The rv SYNTAX score is an exploratory, non-validated metric, it has not yet been externally validated, its interobserver variability has not been assessed, and no clinical association was found in this study. In addition, the small number of outcome events results in low events per variable. Hence, these findings are hypothesis-generating, which also require validation in larger, adequately powered prospective cohorts.

## Conclusion

The interteam agreements on revascularization strategy improved modestly after CMR data were incorporated and were non-uniform across different treatment strategies, with a predominant shift toward less invasive strategies. Although the baseline SYNTAX score and severe LV dysfunction affected the occurrence of MACE, in exploratory analyses, no statistically significant association of revascularization or residual viable SYNTAX score with MACE was detected. As no clear association could be established with clinical outcomes, these findings require further validation in future studies.

## Data Availability

The raw data supporting the conclusions of this article will be made available by the authors, without undue reservation.
